# H_2_O_2_ Significantly Affects *Larix kaempferi* × *Larix olgensis* Somatic Embryogenesis

**DOI:** 10.3390/ijms25010669

**Published:** 2024-01-04

**Authors:** Junjie Zhu, Kaikai Zhang, Huiru Xiong, Yunhui Xie, Rui Li, Xinru Wu, Yun Yang, Hua Wu, Zhaodong Hao, Xiaomei Sun, Jinhui Chen

**Affiliations:** 1 State Key Laboratory of Tree Genetics and Breeding, Co-Innovation Center for Sustainable Forestry in Southern China, Nanjing Forestry University, Nanjing 210037, China; zhujunjie19981009@njfu.edu.cn (J.Z.); xhuir18248703292@sina.com (H.X.); lrshiguang@njfu.edu.cn (R.L.); wxr0410@outlook.com (X.W.); yunyang@njfu.edu.cn (Y.Y.); whua55@126.com (H.W.); haozd@njfu.edu.cn (Z.H.); 2 Key Laboratory of Forest Genetics and Biotechnology of Ministry of Education, Nanjing Forestry University, Nanjing 210037, China; 3 State Key Laboratory of Tree Genetics and Breeding, Key Laboratory of Tree Breeding and Cultivation of State Forestry and Grassland Administration, Research Institute of Forestry, Chinese Academy of Forestry, Beijing 100091, China; zhangkaidekai@163.com (K.Z.); xieyh@caf.ac.cn (Y.X.)

**Keywords:** hybrid larch, somatic embryogenesis, H_2_O_2_, DMTU

## Abstract

Larch is widely distributed throughout the world and is an important species for timber supply and the extraction of industrial raw materials. In recent years, the hybrid breeding of *Larix kaempferi* and *Larix olgensis* has shown obvious heterosis in quick-growth, stress resistance and wood properties. However, its growth and development cycle is too long to meet general production needs. In order to shorten the breeding cycle, we have for the first time successfully established and optimized a somatic embryogenesis system for *Larix kaempferi* × *Larix olgensis*. We found that the highest rate of embryonal-suspensor mass (ESM) induction was observed when late cotyledonary embryos were used as explants. The induced ESMs were subjected to stable proliferation, after which abscisic acid (ABA) and polyethylene glycol (PEG) were added to successfully induce somatic embryos. Treatment with PEG and ABA was of great importance to somatic embryo formation and complemented each other’s effect. ABA assisted embryo growth, whereas PEG facilitated the formation of proembryo-like structures. On top of this, we studied in more detail the relationship between redox homeostasis and the efficiency of somatic embryogenesis (frequency of ESM induction). During subculture, we observed the gradual formation of three distinct types of ESM. The Type I ESM is readily able to form somatic embryos. In contrast to type I, the type III ESM suffers from severe browning, contains a higher level of hydrogen peroxide (H_2_O_2_) and demonstrates a decreased ability to form somatic embryos. External treatment with H_2_O_2_ decreased the somatic embryogenesis efficiency of Type I and type III ESMs, or the higher the exogenous H_2_O_2_ content, the lower the resulting somatic embryogenesis efficiency. We found that treatment with the H_2_O_2_ scavenger DMTU (dimethylthiourea) could significantly increase the somatic embryogenesis efficiency of the type III ESM, as a result of a decline in endogenous H_2_O_2_ content. Overall, these findings have contributed to setting up a successful somatic embryogenesis system for larch production.

## 1. Introduction

The genus larch belongs to the pine family. A total of 25 species of larch exists, and they are naturally distributed across temperate mountains and alpine climate zones in the northern hemisphere. Larch are hardy, cold-resistant trees that grow rapidly and are resistant to decay. Larch wood can therefore be used for the construction of bridges and poles for example, while it also serves as a raw material for the wood fiber industry [1]. It dominates the world’s commercial forests and forest resources [2]. Since the discovery of the natural hybrid between *Larix decidua* and *Larix kaempferi* in Europe in 1990, many countries have been working on crossbreeding larch and have introduced interspecific hybrids with clear hybrid advantages [3]. The (*Larix kaempferi* × *Larix olgensis*) hybrid parents show a significant interspecific genetic variation, resulting in a hybrid vigor through combining the excellent traits of both parents, such as rapid growth, stem form, timber quality, as well as cold, disease and rodent resistance [4]. However, due to differences in interspecific flowering times and long fruiting cycles, the efficiency of breeding trait improvement is unfortunately low, making it difficult to achieve trait improvement targets [5]. Moreover, the currently most broadly applied method of larch asexual reproduction is through cuttings, limiting the widespread application of this excellent tree species. Therefore, it is urgent to establish an efficient and stable somatic embryogenesis protocol for hybrid larch. The somatic embryogenesis technique can give rise to a large number of clonal regenerated plants, using only a small amount of high-quality explants such as seeds to achieve rapid expansion of a target species without being restricted by growing seasons [6]. The pathway and molecular mechanism underlying the somatic embryogenesis process in gymnosperms are completely different from those in angiosperms. There is a special cell mass structure present in the embryonic cells of the gymnosperm-ESM. These cells make it so that only with great difficulty can vegetative gymnosperm cells be transformed into embryonic cells [7,8,9]. Somatic embryogenesis is initiated by ESMs, which consist of both highly vacuolated elongated cells as well as rounded cells that have a high number of inclusions. The highly vacuolated long cells are the suspensor cells, while the round cells with inclusions are the embryonic cells. In *Picea asperata*, ESM development is divided into three subsequent stages: ESM I, ESM II and ESM III. ESM I is directly connected by a small cluster of embryonic cells and a single suspensor cell; ESM II contains multiple suspensor cells; while ESM III is characterized by the presence of enlarged embryonic cells [10].

Stress treatment can promote the efficiency of somatic embryogenesis [11]. The addition of 16 mg/L of abscisic acid (ABA) has previously been found to be beneficial to the formation of regenerating plants during *Larix principis-rupprechtii* somatic embryogenesis [12]. The addition of ABA and polyethylene glycol to the culture medium successfully induced somatic cell embryos of *Pinus sylvestris* [13]. When plants are stressed, they produce reactive oxygen species via their mitochondria, chloroplasts or peroxisomes [14]. Reactive oxygen regulates the expression of embryogenesis-related genes by affecting cell signaling molecules [15]. Studies have shown that controlling oxidative stress to prevent oxidative damage and maintain cell and protein structure integrity is key to larch somatic embryogenesis [16]. However, excessive reactive oxygen species can cause the peroxidation of plant cell membranes, which can be detrimental to the plant [17]. Hao et al. found that the phytosulfokine (PSK)-mediated inhibition of redox reactions in *Cunninghamia lanceolata* promoted somatic embryogenesis [18]. Somatic embryogenesis frequency was found to be positively correlated to antioxidant enzyme activity by adding substances that inhibit antioxidant enzyme activity in a *Lycium chinense* somatic embryogenesis assay [19]. These findings imply that antioxidant activity plays an important role in regulating somatic embryo development.

Although in a number of conifer species, progress has been made regarding somatic embryogenesis research, only a handful of somatic embryogenesis systems, those being *Picea abies* [20,21], *Pinus banksiana* [7] and *Pinus radiata* [22], can be applied for practical production. The stability of somatic embryogenesis systems of most conifer species is very poor. In this study, we established an efficient somatic embryogenesis system using immature (*Larix kaempferi* × *Larix olgensis*) zygotic embryos as explants. We describe the complete developmental process, from ESM induction to the formation of somatic embryos, as well as the effect of PEG or ABA application on somatic embryogenesis efficiency. One of our key findings is that carefully managing H_2_O_2_ content in hybrid larch can improve the efficiency of somatic embryogenesis.

## 2. Results

### 2.1. Establishment of a Somatic Embryogenesis System in Hybrid Larch

**Figure 1:**
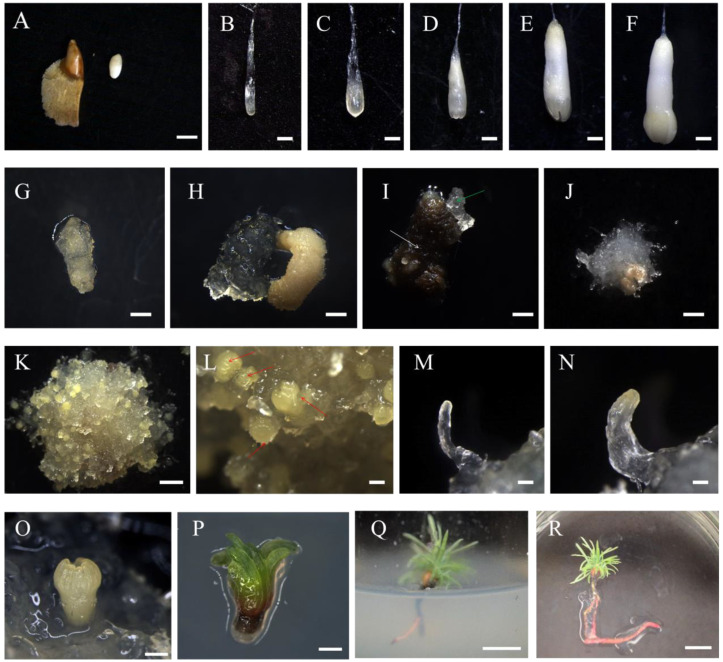
Developmental stages of zygotic (**A**–**F**) and somatic (**G**–**R**) embryos in hybrid larch. (**A**) Hybrid larch immature seeds. (**B**–**F**) Hybrid larch zygotic embryos at different developmental stages for induction of ESM: (**B**) proembryo, (**C**) columnar embryo, (**D**) pre-cotyledonary embryo, (**E**) intermediate cotyledonary embryo, (**F**) late cotyledonary embryo. (**G**–**J**) Induction process of embryonal-suspensor mass (ESM). (**I**) NEC (white arrowhead), ESM (green arrowhead). (**K**) Hybrid larch somatic embryo. (**L**) Somatic embryo (red arrowhead). (**M**–**O**) Stages of somatic embryo development: (**M**) proembryo, (**N**) columnar embryo, (**O**) mature cotyledonary embryo. (**P**–**R**) Germination and plant regeneration. Scale bars: (**A**) = 1 cm, (**B**–**F**) = 2 mm, (**G**–**K**) = 1.5 mm, (**L**–**P**) = 1 mm, (**Q**) = 1 cm and (**R**) = 2 cm.

**Figure 2:**
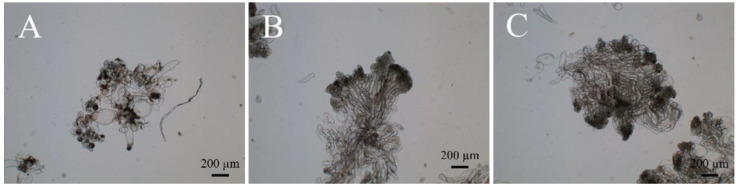
Hybrid larch embryonal-suspensor mass and its microstructure. (**A**–**C**) Microstructures of hybrid larch embryonal-suspensor mass at ESM I, II and III, respectively, with distances measured as shown in the figure.

We performed the induction of ESMs using immature zygotic embryos as explants on DCR medium containing 2.0 to 10.0 mg/L 2,4-D. When zygotic embryos were placed onto the culture medium, they started to expand within 2–4 weeks with ESM structures beginning to appear, yet showing gradual signs of browning (Figure 1G–I). After 3 months, we found that white, transparent, crystal-like ESMs, carrying filamentous projections on their surface, were formed inside the browned ESM (Figure 1I,J). Under the microscope, we observed ESMs with a suspensor structure (Figure 2A–C). The newly formed ESM generally consisted of several tightly arranged, rounded embryogenic cells and a vacuolized, elongated suspensor cell (ESM I stage) (Figure 2A). After being transferred to a proliferation medium for about 15 days, the ESM further proliferated and differentiated, producing more embryogenic cells and suspensor cells (ESM II stage) (Figure 2B). Following another 10 days of growth, the polarity of the ESM was disturbed, becoming a cytoplasmic dense cell mass that showed stable proliferation (ESM III stage) (Figure 2C).

We found significant differences in ESM induction rates for zygotic embryos originating from different collection periods (Table 1). The highest rate of ESM induction was 2%, using embryos harvested 76 days after pollination (late cotyledon embryo stage) (Figure 1F); the second highest rate was 0.8% at 69 d after pollination (middle stage cotyledonary embryo) (Figure 1E); while ESM structures could not be induced in zygotic embryos collected 30 days (proembryo) (Figure 1A), 41 days (columnar embryo) (Figure 1B) and 59 days (early cotyledonary embryo) after pollination (Figure 1C; Table 1).

After transferring the ESM to the somatic embryo induction medium, 15 days later, proembryos with a transparent body and yellowish tip could be observed (Figure 1M). After culturing the ESM on medium for about 30 days, the most apical part of the somatic embryo became bright yellow and opaque, while the embryo volume increased as a whole, forming a columnar embryo (Figure 1N). The embryo apical part then differentiated into a cotyledonary primordium, which subsequently expanded to form a mature cotyledon (Figure 1O). The entire developmental process lasted for approximately 45 d (Figure 1K,L).

We then placed mature cotyledon embryos on the DCR medium, without an osmotic agent, to induce their germination. Their cotyledons began to turn green after 7 days of light, and true leaves developed after 1 month (Figure 1P). At this time, they were transferred to rooting medium containing IBA and NAA, continuing cultivation for 60 days, during which time plants could develop up to 5–6 cm in length (Figure 1Q,R).

### 2.2. ABA and PEG Are Important Stress Signals for Somatic Embryo Development

**Figure 3:**
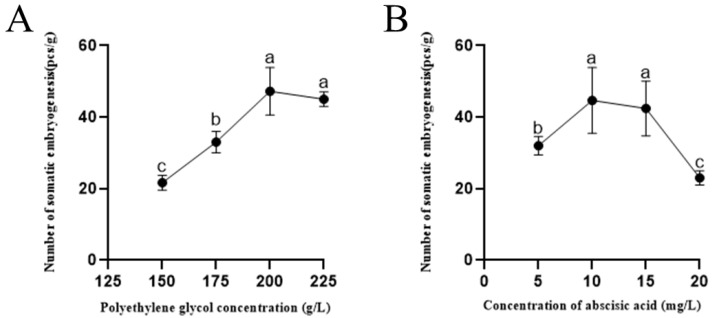
(**A**) Frequency of somatic embryogenesis at different PEG concentrations. (**B**) Frequency of somatic embryogenesis at different ABA concentrations. Data are mean ± SD of three replicates. Duncan’s test was used and different lowercase labels represent significant differences between concentrations (*p* < 0.01).

A high medium osmotic pressure is necessary for somatic embryogenesis in gymnosperms. PEG is the most commonly used osmotic agent during somatic embryo induction in conifers. We compared the effects of different concentrations of PEG (150–225 g/L) on somatic embryo maturation. The number of somatic embryos induced in hybrid larch was found to increase and then decrease as a consequence of elevated PEG concentration (Figure 3A). Analysis of variance (ANOVA) showed that the effect of PEG concentration on the number of induced somatic embryos was highly significant (*p* < 0.01). Additionally, the number of somatic embryos peaked at PEG concentrations of 200 and 225 g/L.

Abscisic acid, the plant “stress hormone”, is widely used in SE induction [23]. In order to elucidate the roles of osmotic stress and exogenous ABA in embryonic development, we compared the effects of different concentrations of ABA (5–20 mg/L) on somatic embryo maturation. We found that the highest number of somatic embryos appeared with the addition of ABA at a concentration of 10 mg/L, followed by ABA at 15 mg/L (Figure 3B). An ANOVA showed that the effect of ABA concentration on the number of somatic embryos was highly significant (*p* < 0.01).

**Figure 4:**
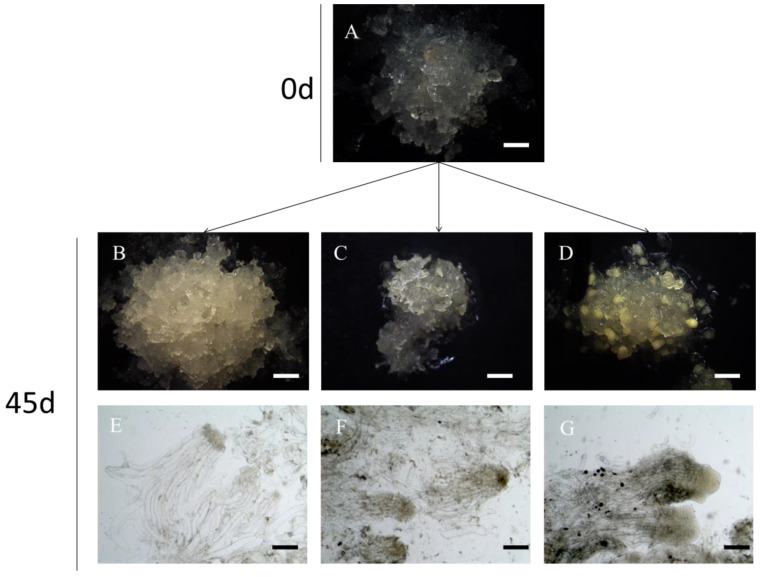
(**A**) ESM in proliferative culture; (**B**–**G**) ESM (upper panel) and its microstructure (lower panel) 45 days after the addition of ABA (**B**,**E**), PEG (**C**,**F**) and PEG and ABA (**D**,**G**). Scale bars: (**A**–**D**) = 1.5 mm; (**E**–**G**) = 200 μm.

To better understand the roles of PEG and exogenous ABA during embryo development, we compared the effect of PEG-containing, ABA-containing as well as both PEG- and ABA-containing medium on somatic embryo development (Figure 4). On the medium containing only ABA, the ESM continued dividing with their differentiation being inhibited (Figure 4B); the embryonic cells stopped developing and became dispersed and vacuolated, and no clusters of suspensor cells were associated with embryo formation (Figure 4E). On the medium containing only PEG, ESM proliferation was inhibited, after which differentiation would be initiated, showing a clear polarity of ESM III. Without the addition of ABA, the ESM stagnated at the proembryo stage, unable to carry out subsequent developmental steps (Figure 4C). At this time, the embryonic cells and suspensor cells were arranged tightly (Figure 4F). Solely on the medium containing both PEG and ABA could we obtain normally developed cotyledonary embryos (Figure 4D), with the apical part of the embryonic cells gradually differentiating into the shape of a cotyledon, and the suspensor cells being closely arranged and elongating continuously (Figure 4G). In conclusion, we found that ABA plays an important role in promoting development, while PEG regulates the shift in the ESM from division to differentiation. ABA and PEG promote somatic embryo formation in unique and complementary ways.

### 2.3. Carbon Source Effect on Somatic Embryo Development

**Figure 5:**
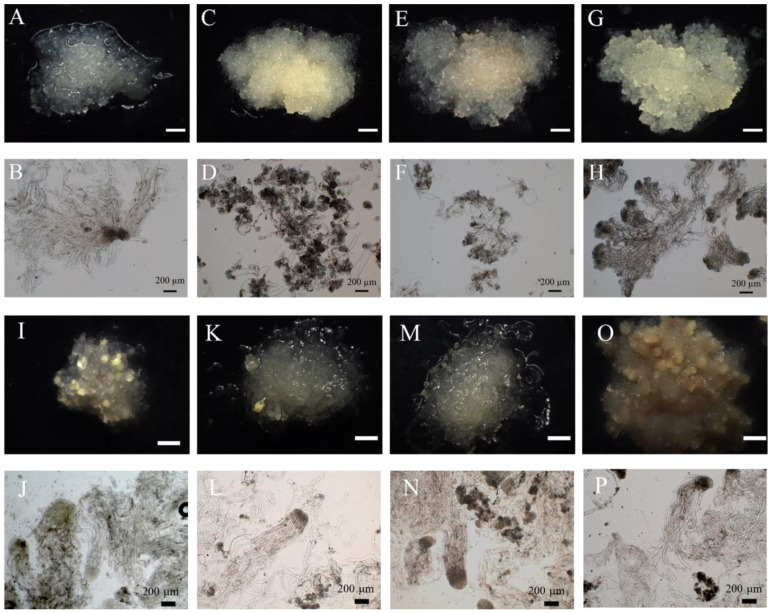
(**A**–**H**) ESM (upper panel) and its microstructure (lower panel) 21 days after the addition of a specific carbon source. (**I**–**P**) ESM (upper panel) and its microstructure (lower panel) 45 days after the addition of a specific carbon source. Carbon sources used: maltose (**A**,**B**,**I**,**J**), sucrose (**C**,**D**,**K**,**L**), fructose (**E**,**F**,**M**,**N**) and glucose (**G**,**H**,**O**,**P**). Scale bars: (**A**,**C**,**E**,**G**,**I**,**K**,**M**) = 1.5 mm, (**O**) = 2 mm, (**B**,**D**,**F**,**H**,**J**,**L**,**N**,**P**) = 200 μm.

We discovered significant differences in the ESM developmental process of hybrid larch in the presence of different carbon sources. When fructose and glucose were used as carbon sources, the structure of the ESM was poorly integrated and dispersed, delaying the development of the ESM at the ESM I stage. Its structure consisted of several rounded embryonic cells and an elongated suspensor cell (Figure 5E–H). When sucrose was used as the carbon source, the ESM became light yellow, with a granular surface, and its structure consisted of dense embryonic cells and several elongated suspensor cells at the ESM II developmental stage (Figure 5C,D). When maltose was used as the carbon source, the ESM was translucent, carrying spines on its surface, with its structure resembling that of the ESM III stage. The ESM embryonic cells showed clusters of rounded cytoplasmic cells, and elongated suspensor cells were distributed around the embryo, a structure that is closer to the white, transparent and viscous ESM of gymnosperms (Figure 5A,B).

**Figure 6:**
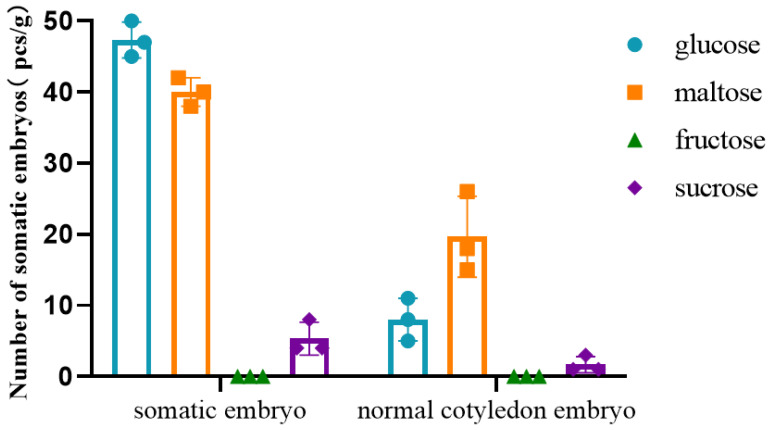
Number of somatic embryos and number of intact cotyledon embryos developing using different carbon sources, data represent the mean ± SD of three replicates.

The selection of the carbon source has a large influence on the process of somatic embryo maturation (Figure 5 and Figure 6). Using glucose as the carbon source led to the most rapid somatic embryo maturation with the highest somatic embryogenesis efficiency. However, on the glucose medium, the vast majority of somatic embryos were deformed and could not develop into regenerated plants (Figure 5O,P and Figure 6). Using maltose as the carbon source led to the second-highest somatic embryogenesis efficiency, but with a much lower percentage of deformed embryos (Figure 5I,J and Figure 6); using sucrose resulted in the formation of a low number of somatic embryos (Figure 5K,L and Figure 6), while using fructose as a carbon source resulted in somatic embryos not forming (Figure 5M,N and Figure 6).

In summary, maltose is the most suitable carbon source to use during ESM proliferation and somatic embryo maturation.

### 2.4. Effects of ROS Homeostasis on Somatic Embryogenesis in Larch

**Figure 7:**
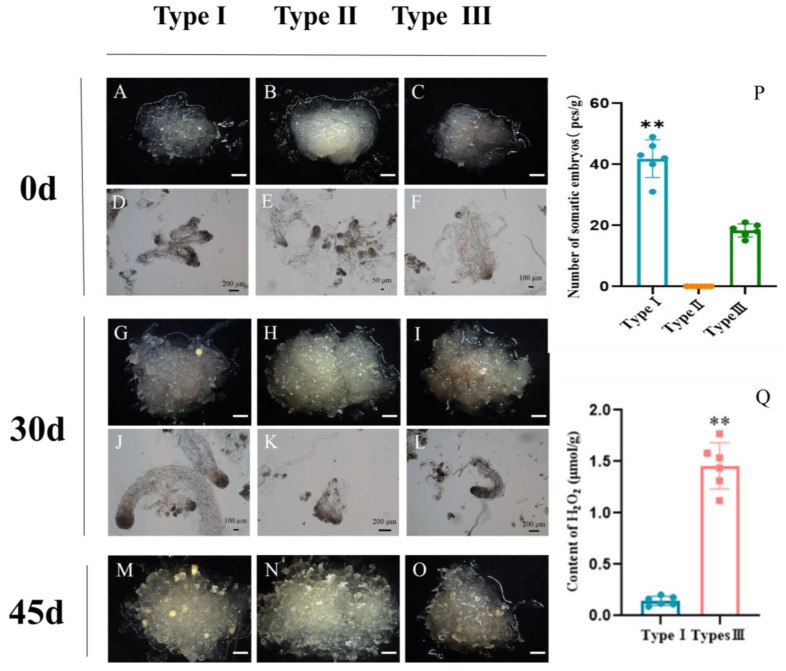
(**A**–**F**) ESM (upper panel) and its microstructure (lower panel) of types I (**A**,**D**), II (**B**,**E**) and III (**C**,**F**). (**G**–**L**) ESM (upper panel) and its microstructure (lower panel) 30 d after somatic embryo induction from ESM of types I (**G**,**J**), II (**H**,**K**) and III (**I**,**L**). (**M**–**O**) ESM 45 d after somatic embryo induction of types I (**M**), II (**N**) and III (**O**). (**P**,**Q**) Bar plots showing the number of somatic embryos produced by different types of ESM (**P**) and H_2_O_2_ content in hybrid larch (**Q**). The error bars indicate the standard deviation between biological replicates (*n* = 6). The comparisons between these three experimental types were separately performed using Student’s *t*-test. ** *p* < 0.01. Scale bars: (**A**–**C**), (**G**–**I**) and (**M**–**O**) = 1.5 mm; (**D**–**F**) and (**J**–**K**) = as shown in the panel.

With a prolonged subculture, the hybrid larch ESM gradually evolved into three distinct types. The Type I ESM appeared translucent with an obvious ESM structure (Figure 7A,D); the type II ESM had a smooth surface without protrusions and scattered suspensor cells (Figure 7B,E); and the type III ESM showed a slight browning (Figure 7C,F). A period of 45 d after somatic embryo induction, an average of 41.83 somatic embryos with mature cotyledons were produced on the type I ESM surface (Figure 7M); the type II ESM (Figure 7N) showed only bumps on its surface with no mature somatic embryos growing out; while the type III ESM (Figure 7O) produced an average of 18.33 somatic embryos. Statistical analysis confirmed that the ESM status has a significant effect on somatic embryo induction in hybrid larch. In summary, the suitability for somatic embryogenesis of the three types of ESM is, in order of decreasing suitability, type I > type III > type II (Figure 7P).

Compared to the type I ESM, the type III ESM not only underwent severe browning, but also showed greatly reduced somatic embryogenesis efficiency. Using a hydrogen peroxide content assay, we found that the hydrogen peroxide content of the type III ESM was much higher than that of the type I ESM (Figure 7Q). We speculated that the difference between the two types of ESM might be due to an unequal H_2_O_2_ content, resulting in differing redox potentials of the two types of ESM.

**Figure 8:**
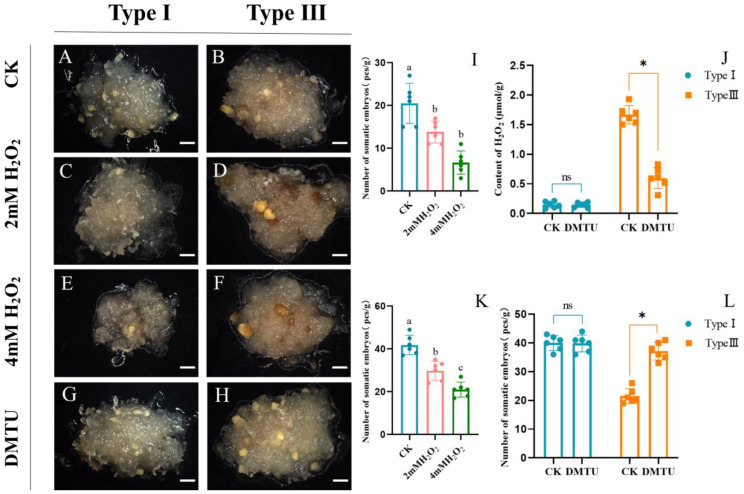
(**A**–**F**) Somatic embryogenesis from type I (**A**,**C**,**E**) and type III (**B**,**D**,**F**) ESM at H_2_O_2_ concentrations of 0, 2 and 4 mM, respectively. (**G**,**H**) Somatic embryogenesis from type I (**G**) and type III (**H**) ESM treated with DMTU. (**I**,**K**) Bar graphs showing the number of somatic embryos developing from type I (**I**) and type III (**K**) ESM at different H_2_O_2_ concentrations. Duncan’s test was used and different letters indicate significant differences (*p* < 0.05). (**J**,**L**) Bar graphs showing the number of somatic embryos (**L**) and H_2_O_2_ content (**J**) after the addition of DMTU to type I (blue) and type III (orange) ESM. Error bars indicate the standard deviation between biological replicates (*n* = 6). Comparisons between these two experimental types were made using Student’s *t*-tests, respectively. ns indicates no significant difference, * *p* < 0.05. Scale bars: (**A**–**H**) = 1.5 mm.

In order to further investigate the effect of H_2_O_2_ on somatic embryogenesis in larch, we used type I and type III ESMs as the starting material for somatic embryo induction, using a medium supplemented with different concentrations of H_2_O_2_. We found that the number of somatic embryos derived from the type I ESM in the control group was 41.83/g, while adding 2 mM H_2_O_2_ reduced that number to 29.83/g with a slight ESM browning (Figure 8A,C,K), and adding 4 mM H_2_O_2_ further reduced the number of embryos to 22.67/g with serious local ESM browning (Figure 8E,K). The ANOVA results confirmed that the number of somatic embryos developing from the type I ESM decreased significantly with increasing H_2_O_2_ concentration (Figure 8K). The number of somatic embryos developing from the type III ESM similarly decreased from the original 18.33/g to 13.83/g and 6.67/g after 2 mM and 4 mM H_2_O_2_ treatment, respectively (Figure 8B,D,F,I). The ANOVA results again confirmed that the number of somatic embryos developing from the type III ESM significantly reduced after H_2_O_2_ treatment (Figure 8I). Therefore, a high H_2_O_2_ concentration reduces the larch ESM somatic embryogenesis efficiency and leads to ESM browning.

To verify the effect of endogenous H_2_O_2_ on somatic embryo induction, we added the H_2_O_2_ inhibitor DMTU to the culture medium and measured the somatic embryogenesis efficiency starting from the type I and type III ESM (Figure 8). An ANOVA showed that DMTU did not affect somatic embryogenesis efficiency when starting from the type I ESM (Figure 8A,G,L), yet significantly increased the number of somatic embryos when using the type III ESM as the starting material (Figure 8B,H,J,L). Combined with the fact that the H_2_O_2_ content of the type III ESM was significantly higher than that of the type I ESM, we can conclude that DMTU promotes larch somatic embryogenesis by reducing the H_2_O_2_ content to tolerable levels.

## 3. Discussion

### 3.1. Establishment of a Hybrid Larch Somatic Embryogenesis System

Somatic embryogenesis in hybrid larch has the potential to provide a strategic reserve of industrial timber resources and large-scale breeding of superior seedlings. It is also an ideal model for studying the mechanisms of embryonic development because of the high similarity between somatic embryogenesis and zygotic embryogenesis [24]. The development of somatic embryogenesis in larch has been underway for many years; however, reports on hybrid larch with a clear hybrid advantage are less frequent. At the same time, there are two main limitations for the large-scale reproduction of hybrid larch by somatic embryogenesis: (1) an extremely low induction rate and (2) a low maturation rate of somatic embryos in many embryogenic lines [25].

Selecting an explant of the correct type and developmental stage is an important factor for the success rate of conifer ESM induction. At present, except for a few studies on using needles [26] and shoots [27] as explants for somatic embryogenesis, the process is usually initiated with immature zygotic embryos serving as the starting material. Immature zygotic embryos are more likely to induce somatic embryos due to their lower degree of differentiation. Zhou et al. found that the induction rate of ESMs in zygotic embryos at different developmental stages of *Cunninghamia lanceolata* gradually increased with an increasing zygotic embryo maturity [24], and ESM induction experiments on *Larix sibirica* zygotic embryos revealed that the highest induction efficiency was achieved with cotyledon initiation [28]. The use of immature zygotic embryos from *Larix gmelinii* [29] and *Larix principis-rupprechtii* [5] was also successful in obtaining ESMs, but the developmental period of immature zygotic embryos that is most suitable for inducing ESMs has not been clearly reported. In our study, only the mid-cotyledonary embryo and the late cotyledonary embryo were capable of inducing ESMs, and the reason for this remains to be investigated as the induction rate of ESMs gradually increased with increasing maturity of the cotyledonary embryo. We can take advantage of this characteristic of hybrid larch, and thus select the period of zygotic embryo development with the highest induction rate for experimentation, in order to obtain more embryonic lineages.

Stress conditions can inhibit cell elongation and alter the state of cell differentiation, which in turn leads to changes in plant cell morphology and induces the formation of somatic embryos [30]. In conifers, the combined treatment of ABA and PEG has become the most commonly used method to induce somatic embryogenesis [23]. PEG can promote the development of ESMs by simulating drought conditions and preventing the formation of aberrant embryos [24], and 15% PEG effectively improves the rate of maturation and quality of somatic embryos in *Swietenia mahagoni* [31]. ABA promotes the maturation of somatic embryos and inhibits their premature germination [32]. The induction of somatic embryos in *Pinus sylvestris* is usually stimulated by the removal of the growth hormone and the addition of PEG and ABA for stress [13], thus promoting the differentiation of somatic cells in the direction of embryonic cells. Exogenous addition of ABA also promotes the synthesis of carbohydrates, lipids and proteins during somatic embryogenesis in coniferous species [33] and prevents precocious development of the embryo [34], while promoting the maturation and differentiation of the somatic embryo during the later stages of development in *Pinus strobus* and *Cunninghamia lanceolata* [24,35]. In addition to ABA’s role in the abiotic stress response [36], changes in the level and expression of related genes influence the capacity for somatic embryogenesis [37]. Three HSP genes expressed during somatic embryogenesis were identified in *Picea glauca* and their expression was found to be induced by ABA [38]. In our study, we used ABA-, PEG- and combined treatments of both ABA and PEG, with the results showing that in the induction medium containing only ABA, ESM differentiation was inhibited. It was clear that ABA did not regulate the early development of the embryo, while PEG regulated the transformation of ESMs into somatic embryos and promoted the development of ESMs, but could not regulate the later developmental steps of the embryo. Complete cotyledonary embryos formed only as a result of the combined treatment with both PEG and ABA. The effects of PEG and ABA on somatic embryogenesis are highly significant, and the two are indispensable to promote the formation of proto-embryos in different ways, probably in an interactive manner.

### 3.2. Homeostasis of Reactive Oxygen Species Affects Somatic Embryogenesis

Dimethylthiourea (DMTU) acts as an H_2_O_2_ scavenger [39] that plays a crucial role in the antioxidant defense mechanism of plants [40]. Previous studies found that appropriate amounts of hydrogen peroxide are involved in signaling and regulating gene expression during plant somatic embryogenesis [41]. The endogenous H_2_O_2_ content in the embryogenic callus in *Gossypium* was shown to be 2.7 times higher than in the non-embryogenic callus and remained higher during early somatic embryo differentiation [42], while exogenous H_2_O_2_ treatment can promote somatic embryogenesis in *Medicago sativa* [43,44].

In this study, the treatment of two different types of ESM with H_2_O_2_ revealed that the number of somatic embryos formed gradually decreases as the concentration of H_2_O_2_ increases. Furthermore, after lowering the endogenous H_2_O_2_ using DMTU, we found that there was no significant change in the number of somatic embryos in the type I ESM, yet a significant increase could be seen in the type III ESM. Since the type I ESM has a lower H_2_O_2_ content than the type III ESM, we concluded that DMTU promotes somatic embryogenesis by reducing the ESM H_2_O_2_ content to physiologically tolerable levels. Stress treatments during somatic embryogenesis can induce stress responses in plants, which can trigger a series of physiological and biochemical changes within the plant. However, due to the limited stress capacity of plants when their endogenous H_2_O_2_ concentration is too high, excessive stress responses can lead to browning of the plant, accompanied by lipid peroxidation, enzyme inactivation and nucleic acid damage [45,46], all of which are extremely detrimental to plant growth. Therefore, we suggest that H_2_O_2_ can promote or inhibit somatic embryogenesis by regulating the expression of somatic embryogenesis-related genes through signaling functions.

## 4. Materials and Methods

### 4.1. Plant Materials

The materials used in this experiment were obtained from hybrid larch seed gardens (*Larix kaempferi* × *Larix olgensis*) located on the Dagujia Forestry farm in Qingyuan County, Liaoning Province. Immature zygotic embryos were collected on 31 May (about 30 d after pollination), 10 June, 28 June, 8 July and 15 July 2020. The collected macrospore balls were sealed in plastic sealing bags on-site and then stored in ice boxes and transported to the laboratory for storage in a 4 °C refrigerator.

Seeds with immature zygotic embryos of different developmental stages were used as the initial explants to induce somatic embryos. The developmental stage of the obtained zygotic embryo material was identified from 5 to 6 randomly selected seeds of each cone according to Shi et al. [47]. Cones were opened and seeds were collected before sterilization. The seeds were washed with detergent for 10 min and then rinsed under running tap water for 30 min to remove residual detergent. Under aseptic conditions, the washed seeds were transferred to autoclaved conical flasks. They were soaked in 75% ethanol for 30 s, sterilized with 10% NaClO for 15 min, rinsed 3~4 times using sterile water, placed on sterile filter paper and blotted dry, removing the seed wings and seed coats as previously described [48]. The embryo sacs, including the zygotic embryos, were used for induction of ESM. More than 200 seeds of the same embryonic stage were used to assess outgrowth frequency and this test was repeated three times.

### 4.2. Medium and Culture Conditions

#### 4.2.1. ESM Induction

The embryo sacs were cut near the suspensor and initially cultured for 3 months at 23 °C in darkness on ESM induction medium. The ESM induction medium consisted of DCR medium [49], supplemented with 2–10 mg/L 2,4-dichlorophenoxyacetic acid (2,4-D), 0.5 mg/L 6-benzyladenine (6-BA), 0.5 mg/L Kinetin (KT), 20 g/L maltose, 450 mg/L glutamine, 500 mg/L casein hydrolysate (CH) (Sigma, St. Louis, MO, USA), 100 mg/L inositol and 2.4 g/L gellan gum (Sigma, St. Louis, MO, USA). The pH of the medium was adjusted to 5.8 ± 0.1 with NaOH or HCl, after which it was autoclaved at 121 °C for 20 min.

#### 4.2.2. ESM Proliferation

ESM subculture medium consisted of DCR medium, supplemented with 1 mg/L 2,4-D, 0.5 mg/L 6-BA, 0.5 mg/L KT, 20 g/L maltose, 450 mg/L glutamine, 500 mg/L CH, 100 mg/L inositol, 2.5 g/L activated carbon and 2.4 g/L gellan gum. The pH of the medium was adjusted to 5.8 ± 0.1 with NaOH or HCl, after which it was autoclaved at 121 °C for 20 min.

#### 4.2.3. Somatic Embryo Induction

After 25 d of stable ESM proliferation, they were transferred to somatic embryo induction medium, which consisted of DCR medium supplemented with 5–20 mg/L abscisic acid (ABA) (Sigma), 5 mg/L gibberellin (GA), 170–250 g/L PEG 8000, 5 g/L inositol, 500 mg/L CH, 450 mg/L glutamine, 200 mg/L aspartic acid, 200 mg/L proline, 25 g/L maltose, 2.0 g/L activated carbon and 2.8 g/L gellan gum. The pH of the medium was adjusted to 6.0 ± 0.1 with NaOH or HCl. ABA was filter-sterilized and added into autoclaved cooled medium.

#### 4.2.4. Plant Regeneration

After 45 days of somatic embryo induction and development, to stimulate germination, mature cotyledon somatic embryos were inoculated on DCR medium without exogenous hormone for the duration of one week. Subsequently, they were transferred to DCR medium supplemented with 0.5–1.5 mg/L IBA and 0.1–0.5 mg/L NAA and maintained at 25 °C under cool white fluorescent light (30 μmol m^−2^ per second, with a 16 h photoperiod) to induce root formation.

### 4.3. PEG and ABA Effect on Somatic Embryogenesis

After 21 days of subculture, all ESMs of an identical, suitable growth stage (~0.2 g in weight) were placed onto somatic embryo induction medium containing either both PEG and ABA, only PEG (150, 175 and 200 g/L) or only ABA (5, 10, 15 and 20 mg/L) to induce somatic embryos. The pH was maintained at 6.0 ± 0.02, and the somatic embryos were cultured for 45 days at 23 °C in the dark.

### 4.4. Carbon Source Effect on Somatic Embryogenesis

ESMs of a similar growth stage (~0.2 g in weight) were inoculated in subculture medium supplemented with different carbon sources (maltose, fructose, sucrose or glucose at a concentration of 20 g/L) for 21 days. Each dish was inoculated with 8 pieces and then placed in an incubator and cultured at 23 °C in the dark. There were 3 replicates in each group.

ESMs of a similar growth stage (~0.2 g in weight) were inoculated in somatic embryo induction medium supplemented with different carbon sources (maltose, fructose, sucrose or glucose at a concentration of 25 g/L) for 45 days. Each dish was inoculated with 8 pieces and then placed in an incubator and cultured at 23 °C in the dark. There were 3 replicates in each group.

Somatic embryo induction medium: DCR medium + 10 mg/L ABA + 200 g/L PEG + 5.0 GA + 0.5 g/L CH + 5 g/L inositol + 450 mg/L glutamine + 25 g/L carbon source + 2.0 g/L Ac + 2.8 g/L Crystal Dish; the pH value was 6.0 ± 0.02.

### 4.5. Hydrogen Peroxide Content Detection

Endogenous H_2_O_2_ content was measured by type I ESM and type III ESM with the same growth stage after 21 days of normal subculture. A total of 6 experiments were repeated in each group. The method used to measure H_2_O_2_ content was as follows: An H_2_O_2_ content detection kit was purchased from Beijing Soraibao Technology Co., Ltd., Beijing, China. An amount of 0.1 g of hybrid larch ESM was weighed, 1 mL of acetone was added to an ice bath and the ESM was ground to a homogenate and then transferred to a 1.5 mL centrifuge tube. The sample was centrifuged at 8000× *g* at 4 °C for 10 min, and 250 mL of supernatant was taken, to which 25 μL of titanium sulfate and 50 μL of concentrated ammonia water were added to precipitate out the titanium peroxide complex. The sample was centrifuged at 4000× *g* at room temperature for 10 min, carefully removing the supernatant and washing the remaining precipitate 3–5 times using 5 mL of acetone, after which 250 μL of sulfuric acid solution was added, followed by vigorous shaking to resolubilize the precipitate. The sample was then let to rest at room temperature for 5 min, while the microplate reader was preheated for more than 30 min. An amount of 200 μL of sample was transferred to a 96-well plate to determine the absorbance value at 415 nm, using an H_2_O_2_ standard solution and 2 μmol/mL acetone to determine the standard value and blank control, respectively.

### 4.6. H_2_O_2_ Effect on Somatic Embryogenesis

Type I and type III ESMs of the same growth stage (~0.2 g in weight) were placed on somatic embryo induction medium supplemented with exogenous H_2_O_2_ or DMTU after 21 days of subculture. Each dish was inoculated with 8 pieces and then placed in an incubator and cultured at 23 °C in the dark. A total of 6 experiments in each group were repeated, and the efficiency of somatic embryogenesis was quantified. The DMTU working concentration used was 10 mM, and the H_2_O_2_ working concentrations used were 0, 2 and 4 mM. DMTU and H_2_O_2_ were filter-sterilized and added into autoclaved cooled medium.

### 4.7. Statistical Methods

Regarding the ESM induction assay, each hybrid combination was inoculated with at least 200 seeds with three replicates for each treatment and significant differences were tested using SPSS 23.0.

One-way ANOVA was performed using SPSS23.0 for data analysis. Duncan’s multiple range test was used to compare the mean values, and the significance level was set to α = 0.05. Graph Pad was used to plot the analysis results of SPSS 23.0 software.

Regarding the somatic embryo induction assay, each experimental group was inoculated with at least 5 dishes and 3 replicates per group.

The developmental period of the zygotic and somatic embryos was identified using a somatic microscope (Leica, S8AP0, Leica Microsystems (Switzerland) Ltd., Heerbrugg, Switzerland), and microstructures were observed using a Zeiss inverted microscope (ZEISS, DMI4000, Carl Zeiss AG, Oberkochen, Germany).

## 5. Conclusions

In summary, 2.0 mg/L of 2,4-D- and 0.5 mg/L of BA-supplemented DCR medium can effectively induce ESMs from immature (*Larix kaempferi* × *Larix olgensis*) embryos. These ESMs can successfully initiate somatic embryo differentiation in mature medium supplemented with 200 g/L of PEG and 10 mg/L of ABA. Furthermore, we found that carefully managing the H_2_O_2_ content in hybrid larch can improve the efficiency of somatic embryogenesis. However, the molecular mechanism of promoting somatic embryogenesis by balancing the hydrogen peroxide content still needs further study. In a follow-up study, transcriptome sequencing following DMTU treatment was systematically carried out to identify key gene regulatory networks and metabolic pathways, to find the downstream functional genes, to promote the further improvement in somatic embryogenesis efficiency of hybrid larch, and to lay a foundation for the preservation, development and application of excellent germplasm resources of hybrid larch.

## Figures and Tables

**Table 1:** Effect of explant collection date on ESM induction rate in hybrid larch.

Collection Date	Developmental Period	Inoculation Number	ESM Induction Rate (±SD)/%
30th May	Proembryo	233	0 c
10th June	Columnar embryo	241	0 c
28th June	Early cotyledonary embryo	252	0 c
8th July	Mid-cotyledon embryo	250	0.8 ± 0.53 b
15th July	Late cotyledon embryo	250	2 ± 0.21 a

a–c: Different letters indicate significant differences *p* < 0.05.

## Data Availability

Data will be made available on request.
